# The Voluntary Hospitals and National Insurance

**Published:** 1911-12-16

**Authors:** 


### A SYMPOSIUM OF HOSPITAL MANAGERS.

### The Voluntary Hospitals and National Insurance.

THE present Government have apparently decided to subject the working men of this country, when seriously ill, by State action through the Insurance Bill, to grievous risks and possibly much avoidable suffering from which they have previously been absolutely freed through the voluntary hospitals. We invite hospital managers and the representatives of the workshops which contribute to voluntary hospitals to send us their views on the probable effects of the National Insurance Bill upon:

(*a*) The voluntary hospitals, financially.

(*b*) The amount of the present accommodation for in-patients which the voluntary hospitals may be unable to maintain.

(*c*) The eligibility or otherwise of insured persons to free hospital benefit.

(*d*) What is likely to be the consequence to the working classes of being shut out from in-patient relief at the voluntary hospitals, which they at present enjoy, on the ground stated to the Hospital deputation by Mr. Lloyd George, *i.e.*, that when they are insured they will have no right to come to the hospitals?

We have already received communications on these points, and shall welcome a letter from anyone who has studied the questions involved and has an acquaintance with the facts. In this way we hope to dispel much of the want of knowledge of the facts which exists generally at present, and to induce the workmen throughout the United Kingdom to study these questions. At the same time, we hope to make the position of the voluntary hospitals and their work, as well as the views and zeal of the workmen for these institutions, more thoroughly appreciated and more widely known even than they are at present.

